# Briefly Flashed Scenes Can Be Stored in Long-Term Memory

**DOI:** 10.3389/fnins.2018.00688

**Published:** 2018-10-05

**Authors:** Arnaud Delorme, Marlène Poncet, Michèle Fabre-Thorpe

**Affiliations:** ^1^Centre de Recherche Cerveau et Cognition, Université Toulouse III - Paul Sabatier, Toulouse, France; ^2^Centre National de la Recherche Scientifique, Centre de Recherche Cerveau et Cognition, Toulouse, France; ^3^Institute for Neural Computation, University of California, San Diego, La Jolla, CA, United States; ^4^Institute of Noetic Sciences, Petaluma, CA, United States

**Keywords:** categorization, long-term memory, visual perception, animal images, go/no-go task

## Abstract

The capacity of human memory is impressive. Previous reports have shown that when asked to memorize images, participants can recognize several thousands of visual objects in great details even with a single viewing of a few seconds per image. In this experiment, we tested recognition performance for natural scenes that participants saw for 20 ms only once (untrained group) or 22 times over many days (trained group) in an unrelated task. 400 images (200 previously viewed and 200 novel images) were flashed one at a time and participants were asked to lift their finger from a pad whenever they thought they had already seen the image (go/no-go paradigm). Compared to previous reports of excellent recognition performance with only single presentations of a few seconds, untrained participants were able to recognize only 64% of the 200 images they had seen few minutes before. On the other hand, trained participants, who had processed the flashed images (20 ms) several times, could correctly recognize 89% of them. EEG recordings confirmed these behavioral results. As early as 230 ms after stimulus onset, a significant event-related-potential (ERP) difference between familiar and new images was observed for the trained but not for the untrained group. These results show that briefly flashed unmasked scenes can be incidentally stored in long-term memory when repeated.

## Introduction

Humans can remember thousands of pictures after a single exposure of 5 s (Shepard, 1967; Standing et al., 1970, Standing, 1973). More impressively, recent studies have showed that the representations stored in memory are precise (Hollingworth and Henderson, 2002; Vogt and Magnussen, 2007; Brady et al., 2008). For example, in the study of Brady et al. (2008), after viewing 2500 pictures of objects, participants were shown two images and asked which of the two they had seen in a two-alternative forced choice (2AFC). The new picture could either be an object from a different category, a new exemplar from the same category, or the same object but in a different state. Impressively, participants successfully discriminated between the previously seen object and the new one with 87% accuracy in the state condition. These results demonstrate that humans have a massive and detailed memory capacity for pictures.

In these studies, presentation time of the stimuli was very long (from 3 to 20 s). However, it is well known that recognition memory suffers when stimulus duration decreases (Shaffer and Shiffrin, 1972; Potter, 2012). For example, Potter (1976) showed that it is possible to remember 80% of masked pictures when presented for 120 ms but only 50% if presented for only 50 ms. Other studies argued that scene representations would first be represented as a general layout or gist and details about specific objects would be added on subsequent fixations (Melcher, 2001; Melcher and Kowler, 2001; Tatler et al., 2003). Generally, the amount of details remembered about a stimulus increases linearly with more time to encode them (Brady et al., 2009). Therefore, long enough stimulus duration seems necessary to encode and recall complex visual scenes in details.

Yet, the repetition of briefly presented pictures improves memory performance. In a study by Melcher (2001), participants were presented with scenes including 12 unrelated objects either in one viewing or as repeated brief views (from 0.25 to 2 s) for a cumulative viewing duration of 1–4 s in both conditions. The number of items recalled after 4 s of a continuous presentation was the same as the number of items recalled after 4 trials of 1 s even though each presentation was separated by other stimuli. These results, as well as the one of Martini and Maljkovic (2009) using a RSVP paradigm, are consistent with the Total Time Hypothesis proposing that a fixed amount of time is required to learn a fixed amount of information regardless of how this time is distributed (Bugelski, 1962; Cooper and Pantle, 1967).

On the other hand, the improvement of memory performance thanks to repetition was tested only after a few minutes and might not involve long-term memory but a “medium-term” memory – as referred to by Melcher (2001). Indeed, the accumulation of memory with re-test trials did not extend across separate days (Melcher, 2001; Melcher and Kowler, 2001). Thus the traditional long-term memory was most probably not involved in the increase of performance with repetition. Furthermore, recent results showed that the memory tested after few minutes in rapid serial visual presentation (RSVP) tasks declines quickly with (a) increased time between the stimulus presentation and test and (b) increased number of intervening test pictures (Potter et al., 2002; Endress and Potter, 2012). Therefore, even though repetition might improve recognition performance on a short timescale, repeated brief presentations might not be sufficient to encode images in long-term memory.

The first aim of our study was to test visual recognition memory for complex natural scenes flashed for only 20 ms. To further test the effect of repetition on memory performance, one group of participants saw the to-be-recognized images only once and a second group saw the same images set over several days for a cumulative time of 440 ms. Such short stimulus presentation time has never been used in long-term memory recognition. Indeed, it reduces the stimulus energy and prevents the possibility of making saccades to encode details about the stimuli.

We challenged memory performance on two other aspects apart from the use of extremely short stimulus presentation time. First, participants were not explicitly asked to memorize the set of images and were unaware that they would be tested on a recognition task. Second, instead of a usual 2AFC recognition task, we tested our participants in a go/no-go paradigm. Participants had to decide very rapidly (within 1 s) for each image whether they had seen it earlier (familiar/non-familiar task) without any helps from a distractor image (as it could be the case in a 2AFC). Long-term memory experiments do not usually require participants to answer in a limited time window. For example, in Brady et al. (2008) participants’ reaction times were typically above 2 s.

This type of go/no-go paradigm also allows assessing how storage in long-term memory is influenced by the category an image belongs to in the encoding phase. Is the storage highly dependent on categorical processing (Ranganath et al., 2004) or is it independent of categorical processing. If dependent on categorical processing, one might expect that images belonging to the same category will show lower performance than images belonging to different categories. The relative latency of the implicit image category brain processing during the image recognition phase, may be an indication of the role of the image category in stimulus memory encoding.

We finally investigated a possible correlation between participants’ performance in the recognition memory task and event-related-potential (ERP) measures. Since the study of Rugg et al. (1998) an entire branch of research has studied the mechanisms of recognition memory for words and pictures using ERPs. The main goal of these studies was to differentiate familiarity (unspecific awareness that an item has been encountered) from recollection processes (conscious retrieval of both item and contextual details). In general these studies compared ERPs between previously seen and novel images. They reported two distinct components of this old/new effect: an early (300–500 ms) mid-frontal component related to familiarity based recognition and a late (500–700 ms) parietal effect related to recollection (e.g., Curran and Cleary, 2003; Rugg and Curran, 2007; Zimmer and Ecker, 2010; but see also Voss and Federmeier, 2011 for another interpretation of the familiarity effect). Because the go/no-go task lead to fast reaction times (Thorpe et al., 1996), we expect to see memorization familiarity based recognition at much earlier latencies in our task.

To summarize, we tested along with EEG recordings whether participants could perform a memory recognition task (familiar/non-familiar) on complex images that were flashed only 20 ms in an incidental task. In particular, what are the difference in terms of brain activity between a trained and an untrained group, which would be indicative of long-term memory storage. Our study challenges memory performance on several aspects: incidental learning, very short stimulus presentation time, and fast recognition.

## Materials and Methods

### Participants

The trained group included 14 participants (7 women, mean age 26 ± 1), although EEG data from one subject proved to be too noisy to be analyzed. EEG was recorded in the recognition memory task for only 10 of them. The untrained group included 10 participants (4 women, mean age 25 ± 1) all of them have been tested along with EEG recordings. All participants had normal or corrected to normal acuity and provided written informed consent. This study was carried out in accordance with the recommendations of Toulouse University. The experimental procedures used were authorized by the local ethical committee (CCPPRB No. 9614003).

### Stimuli

**FIGURE 1:**
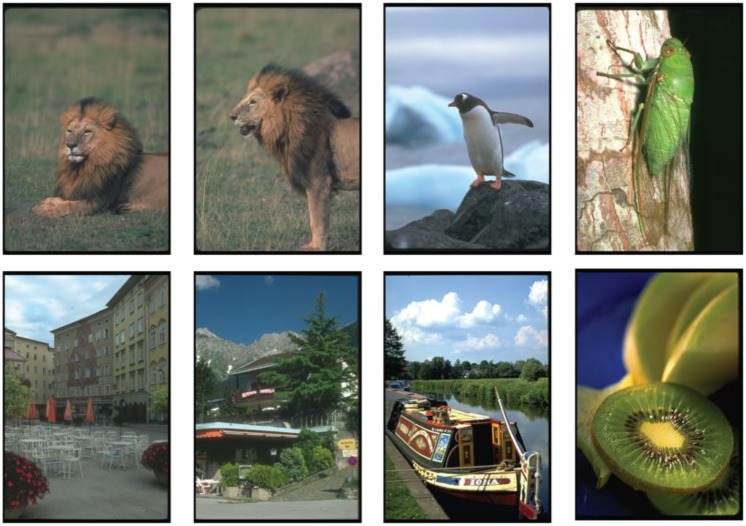
Examples of animal **(Top row)** and non-animal **(Bottom row)** images used in the experiment.

The 2000 images used in the experiment were colored natural images from the Corel database. Half of the images included animals (mammals, birds, insects, etc.), the other half was composed of landscape, fruits, buildings, etc., The range of images was very large but at the same time, some target images could be very similar: different exemplars of the same basic category (e.g., lion) were present in familiar and new images (**Figure 1**). Therefore, having a vague sense of the presence of a lion in the picture would not be enough to distinguish this image from another new image including a lion.

Trained participants were separated in 7 pairs. For each pair, a subset of 200 images, half animals and half non-animals, was randomly picked to be its familiar set and all 200 images were different from the 200 familiar images of another pair (one male one female) of participants (participants were considered in pair so we had 14 subjects and 7 sets of 200 familiar images). This allowed to counterbalance familiar and unfamiliar images across subjects. Images which were familiar for one pair of subject were new for the other pairs of subjects so that a specific set of familiar images could not be responsible for some of the differences observed in the recognition phase. Without this procedure, one might argue that the specific set of familiar images we choose is responsible for some of the differences we observed in the recognition phase. In the recognition task, the 200 images were repeated 3 times (see procedure below) while the new images (600) were never repeated and were the same for all participants – the new images were images that had never been seen by the participants. The same set of familiar and new images presented to an untrained subject corresponded to the ones presented to a trained subject for whom EEG was recorded. Therefore, the image set was the same for the untrained and the trained participants for whom EEG was recorded.

### Procedure

**FIGURE 2:**
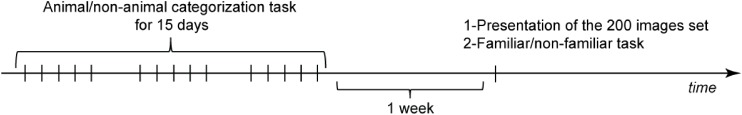
Experimental protocol. The trained group performed a categorization task on the familiar image set for 15 days. After a week both trained and untrained participants saw the familiar image set in the study phase and were then tested on a surprise recognition memory task.

The experiment was composed of three stages: a training phase, a study phase and a test phase (**Figure 2**). The training phase, only performed by the trained group, consisted in an animal/non-animal categorization task on the familiar image set repeated 5 days per week over 3 weeks (for details about each phase, see below). One week after the last training session, both trained and untrained participants saw the 200 familiar images set (study phase). Participants were then asked few minutes later to perform an unexpected recognition test on these images (test stage). This latter task was associated with EEG recordings.

In all three tasks, participants were seated approximately 0.5 m from a 200 Hz cathode ray tube screen. A fixation cross was presented on the center of a black screen for a random time between 800 and 1200 ms. The stimulus (horizontal or vertical) was then flashed for 20 ms (7 × 10° visual angle) corresponding to four frames at 200 Hz. Note that this brief presentation time ensure that even microsaccade, which can occur in the 30 Hz frequency range (Hipp and Siegel, 2013) are not used by the visual system to encode information. At this distance of the screen, visual acuity is about 40% of what it is in the fovea (Kalloniatis and Luu, 2008). At the fovea, visual acuity is considered to be 0.00025 degree visual angle (1 min of arc). The image resolution is presented on a 600 × 800 CRT computer screen at 0.5 m from the subject, which correspond to 0.00075° visual angle for each pixel. It is therefore reasonable to assume that the visual system can process the full resolution of the image as presented on the screen, including small details at the periphery of the image, even with briefly flash stimuli that prevent visual exploration of the images. Participants had to release their finger as fast and as accurately as possible if they saw a target stimulus (animal in the categorization task, familiar image in the recognition task) or as soon as they identified the image (study task). If they did not release their finger for 1 s then the response was considered as either a correct rejection if the image was a distractor or a miss if the image was a target. Go response latencies were recorded via an optical response pad synchronized with the stimulus display.

### Training: Categorization Task

The trained group performed a go nogo animal/non-animal categorization on their 200 familiar images set for 15 days. On the two last days, this short training categorization task was followed by a categorization test. In this test, participants performed an animal/non-animal categorization task over 12 blocks of 100 trials. The familiar image set was repeated three times and mixed with 600 new images. Therefore in total, participants saw their familiar image set 21 times during the training phase. The results of the categorization test showed that 3 weeks training had little effect on categorization performance. Accuracy was slightly better, but the only effect on reaction time was seen on the slowest responses that were speeded-up by training; fastest reaction times did not change (full details about the procedure and the results of this training phase have been published in Fabre-Thorpe et al., 2001). Note that, in this experiment, unlike the trained group, the untrained group never performs the animal/non-animal go nogo categorization task, although some of the participant might have known of the go nogo categorization task since it has often been used in our prior experiments.

### Study: Familiarization With the Image Set

Before the recognition task, both the trained and the untrained group saw their familiar image set once (which was the first time for the untrained group). The trained group was asked to release their finger as soon as they detected a picture whereas the untrained group was asked to release their finger as soon as they identified the stimulus flashed on the screen. This task had different purposes. First it familiarized the untrained participants to the set of stimuli that will be tested in the recognition task. Second, by asking trained participants to release their finger on all images, we tried to reduce the automatic response behavior (response to animal) that they could have learned during the training phase.

### Testing: Recognition Memory Task

Following the study phase, participants of the two groups performed a surprise recognition task. They were asked to release their finger as soon as they felt that they had seen the same picture earlier. They were that about half of the trials contained targets. The 200 familiar image set was mixed with 600 new images (different from the ones used in the categorization test). Participants performed 12 blocks of 100 trials; the familiar image set being repeated once every 4 blocks (3 times in total) but fully randomized within each 4-block series. All images were randomly presented and in each block, 25 images were familiar animal images, 25 were familiar non-animal images, 25 were new animal images and 25 were new non-animal images.

### Behavioral Analysis

Median reaction times (RT) for correct go responses and d’ (Snodgrass and Corwin, 1988) were calculated for each participant in the recognition task and used in statistical analysis. Independent *t*-tests were applied to compare performance between the trained and the untrained group. Because the familiar image set was repeated 3 times over the experiment (once every 4 blocks), we tested any effect of repetition by dividing the results into 3 (4 blocks × 3) and applied a 2-way repeated ANOVA. If an effect was found, *post hoc* analyses using paired *t*-tests between the 1st and 2nd repetition as well as between the 2nd and 3rd repetition were applied. *P*-values are reported after Bonferroni correction for multiple comparisons. Finally, as the status of the image during the training phase (animal target vs. non-animal distractors) could influence recognition, comparisons between animal and non-animal stimuli were done by using paired *t*-tests for each of the 2 groups separately. Results are given with the average ± standard error of the mean (SEM). All behavioral statistics were computed using Statistica (TIBCO Software Inc.) and Statview (Scientific Computing Inc.).

### EEG Recording and Pre-processing

**FIGURE 3:**
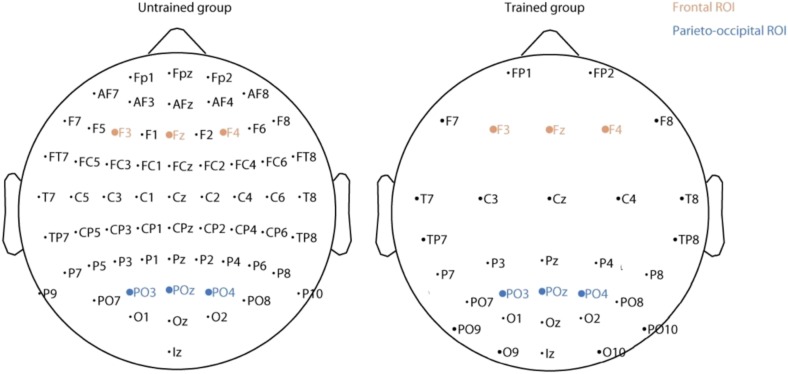
Location of the electrodes for the untrained **(Left)** and trained **(Right)** group. The electrodes included in the two regions of interest are in shown brown (frontal) and blue (parieto-occipital).

For the trained group, EEG activity was recorded using a 32-channel SynAmps amplification system (Neuroscan) using a sampling rate of 1 kHz and linked ears as the reference. For the untrained group, a 64-channel BioSemi system was used, using a sampling rate of 1024 Hz and referenced to Cz (data was re-referenced later to an averaged reference). Because we did not use the same EEG recording montage for both trained and untrained participants, direct comparisons of all the channels between the two groups is not possible (see **Figure 3**). Therefore each EEG analysis was performed for each group separately and we focused on a subset of channels common to both groups. Also, all ERP figures show ERP differences between conditions and such ERP differences are not affected by the choice of reference.

Data pre-processing was conducted using EEGLAB (Delorme and Makeig, 2004). EEG signal was band-pass filtered at 0.2–512 Hz and a 50 Hz notch filter was used. An Independent Component Analysis was applied to identify eye-blinks that were later removed by visual inspection of the Independent Component scalp topographies. In a few subjects, components isolating obvious muscle artifacts and blinks were removed but in general a conservative approach was taken to avoid removing meaningful neuronal activity. Each trial was divided into epochs from −100 to 600 ms relative to stimulus onset. Each epoch was baseline corrected by subtracting the average activity between −100 and 0 ms from each EEG data channel.

### ERP Analysis

For each participant, event related potentials for familiar and for new images was calculated (familiarity effect from the study set). A paired *t*-test was then applied to compare ERPs between these two conditions for each electrode and at each time point. The obtained *p*-values were corrected for multiple comparisons using false discovery rate (FDR) (Bejamini, 2001) estimation both across time and electrodes. FDR was calculated using the fdr.m function of EEGLAB (Delorme and Makeig, 2004). Based on these first results and on previous studies showing the important role of frontal and parietal regions in memory tasks (for a review see Rugg and Curran, 2007), we chose two regions of interest: a frontal region including F3, Fz, F4 and a parieto-occipital region including PO3, POz, PO4 (see **Figure 3**). The topography of the ERP in individual channels supported the selection of frontal and parieto-occipital electrode sites for analyses. Because of volume conduction in EEG electrode and their closest neighbors are strongly correlated. ERP differences between familiar and new images for these two regions were computed for each time point using paired *t*-tests FDR-corrected. The same procedure was used to compare animal and non-animal images.

Because a familiarity effect was observed for the trained group but not for the untrained group, further ERP analyses were only performed for the trained group. The first analysis tested the effect of repetition of the familiar images in the recognition memory task. For the two regions of interest, the familiarity effect was computed for each of the three repetitions. A repeated measures ANOVA was then applied on each time point with FDR-correction. A second analysis tested the effect of stimulus category on the familiarity effect. For each stimulus type (animal or non-animal), an familiarity difference was calculated (ERP familiar animal minus ERP new animal and ERP familiar non-animal minus ERP new non-animal) for the two regions of interest. Paired *t*-tests were then performed with FDR correction.

Studies on recognition memory tasks usually compute ERPs for hits and correct rejections only. However, we decided to use all trials (correct and incorrect responses) and divide the data only depending on the trial condition for several reasons. First, untrained participants performed the recognition task close to chance level (see behavioral results). ERP analyses using only correct trials would have been very noisy since very few trials would have been considered and within these trials, some might be correct even though participants did not recognize the image. By contrast, the trained group exhibited high performance so ERP results would be similar irrespective of the inclusion or exclusion of incorrect trials. Second, including all trials allowed us to test whether the ERP difference between familiar and unfamiliar trials was due to recognition or the repetition of the familiar image set. If a difference is found in both trained and untrained groups then, assuming that untrained group recognition performance will be close to chance expectation, the ERP difference would likely be interpreted as the consequence of the repetition of the familiar stimuli (priming) and not the neuronal correlates of familiarity. Finally, behavioral responses in the comparison of the early ERPs between animal and non-animal images were not relevant, so it was logical to be consistent in our analysis and use all the trials in all of our analyses.

## Results

### Memory for Briefly Flashed Images

**FIGURE 4:**
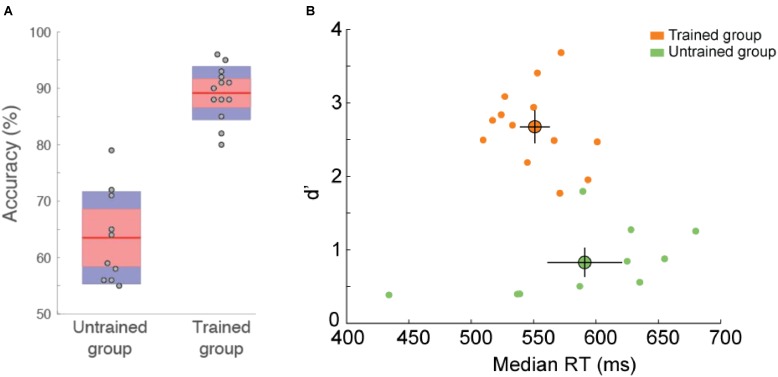
Behavioral performance in the recognition memory task (familiar vs. non-familiar images). **(A)** Accuracy (%correct) for untrained and trained participants. Dots indicate individual subject accuracy. Red region indicate standard deviation and blue region indicate 95% SEM. Error bars show SEM. **(B)** d’ as a function of reaction time. Each dot represents the performance of one participant. Average performance for each group is represented by the large colored dot with SEM.

Recognition performance was higher for trained than for untrained participants [*t*(21) = 8.52, *p* = 3E–8, effect size Cohen’s *d* = 3.72]. Untrained participants were able to recognize 64.5 ± 2.5% of the images (*d* = 0.83 ± 0.15) that they saw in the study phase and trained participants were able to perform the memory recognition task at 88.7 ± 1.5% (*d* = 2.67 ± 0.15), the best participant recognizing 96.5% (*d* = 3.68) of the images (**Figure 4**). This suggests that trained participants stored the briefly flashed familiar image set in long-term memory while they performed the categorization task during the training phase. Although memory encoding might benefit from visual exploration (Glen et al., 2013), we showed that visual exploration is not required to encode complex visual images since such exploration was not possible in this experiment. Furthermore, encoding of the images was done without explicit instruction to memorize them. Since trained participants were not informed of the study and test phase prior to doing it, they did not look for or try to remember features that would enable them to distinguish between two exemplars of the same category (e.g., specific attributes of a familiar lion that would help differentiate it from another lion). The fact they were able to perform the recognition task with high level of accuracy suggest that the implicit representation included many details of the image.

**FIGURE 5:**
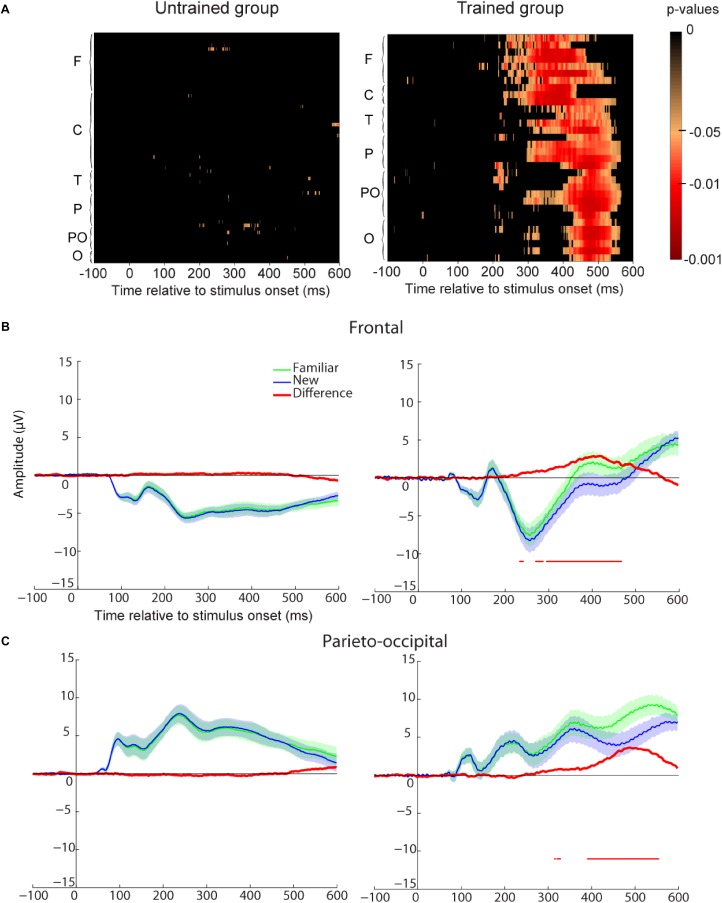
Recognition memory task event-related-potential (ERP) results. **(A)** Paired *t*-test *p*-values false discovery rate (FDR-corrected) for the ERP difference between all familiar and new images and all electrodes. The difference is represented at each time point from **–**100 to 600 ms relative to stimulus onset. Electrodes are grouped by regions based on their first letter (F, frontal for electrodes starting with F and AF; C, central; T, temporal; P, parietal; PO, parieto-occipital; O, occipital with left hemisphere electrodes represented before right hemisphere electrodes and Iz included in this group). Untrained group results are on the **Left** and trained group results are on the **Right**. **(B)** Frontal ROI as defined in **Figure 2**. Average ERP for familiar (green) and new (blue) images. The difference between the two ERPs is represented in red. For each time-point a paired *t*-test was calculated and reported by an horizontal thin red line underneath the ERP if it was significant (*p* < 0.05 FDR-corrected). Shaded areas indicate SEM. **(C)** Parietal ROI. Legend as in **B**.

To assess the neural correlates of familiarity, we compared participants’ ERP for familiar vs. new images for all electrodes at each time point (see method section and **Figure 5A**). We found no statistical difference between these two conditions for the untrained group. However, a significant difference was found for the trained participants starting around 230 ms after stimulus onset. This familiarity effect appeared first in frontal and central regions and later in parieto-occipital and occipital regions. To further investigate the difference between these two regions, we represented ERP for frontal and parieto-occipital regions separately (see method section and **Figures 5B,C**). For the trained group, the familiarity effect was significant from 230 ms after stimulus onset in frontal regions and from 315 or 390 ms in parieto-occipital regions. This dissociation could be interpreted as the familiarity (early) and recollection (late) components observed in other ERP studies (Curran, 2000; Curran and Cleary, 2003; Vilberg et al., 2006).

One could argue that the ERP difference between familiar and new images reflects a difference in motor response (go response for familiar images vs. no-go response for new images). However, motor ERP component is usually located around central or parietal electrodes while in our study the effect is seen in all electrodes. Furthermore, if we compare go responses to no-go responses for the untrained participants, we do not find any difference in the first repetition when performance was at chance expectation (see **Supplementary Figure S1A**) and the motor-related difference was only visible after 500 ms in parietal regions when considering all repetitions (see **Supplementary Figure S1B**). Another possibility could be that the familiarity effect was due to the three repetitions of the images in the experiment (performance and behavior and ERPs are analyzed separately for each repetition in the next section). However, the effect was present for the trained group but not for the untrained group. Therefore, the ERP difference between familiar and new images found for the trained group was most likely related to memory.

### Repetition Effect Within the Experiment

**FIGURE 6:**
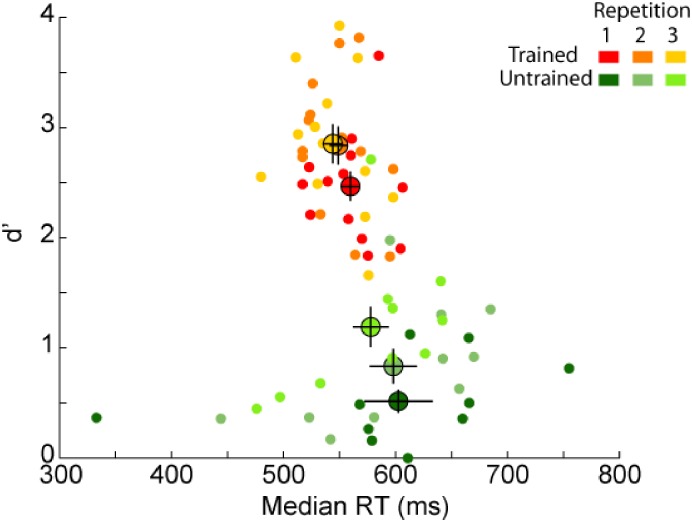
Behavioral results [d’ as a function of reaction times (RT)] for each repetition of the familiar image set. Average performance for each group (trained participants in orange, untrained participants in green) is represented by a bigger dot with SEM. Each dot represents the performance of one participant in each of the three repetitions separately (from dark for the first repetition to lighter colors for the following repetitions). Note that if the majority of untrained participants had lower d’ performance than trained participants, one untrained participant reached similar d’ performance as trained participants in the last repetition.

Because the familiar image set has been used three times during the recognition task, it might be possible that some learning occurred over the experiment. In the procedure, the familiar image set was repeated once every four blocks. Therefore, to assess the effect of learning we compared participants’ performance in each of the three repetitions separately for each of the trained and untrained group (**Figure 6**). Considering the untrained group, RTs did not change over the experiment (2,18) = 1.18, *p* = 0.33, however, we found an effect for d’ [*F*(2,18) = 18.81, *p* = 4E–5, η^2^ = 0.99]. *Post hoc* comparisons showed that d’ increased between both the first and second repetition [*t*(9) = 2.92, *p* = 0.03; *d* = 1.95] and the second and third repetition [*t*(9) = 6.01, *p* = 4E–4, *d* = 4]. Considering the trained group, we found an effect of the familiar image set repetition for RTs [*F*(2,24) = 6.98, *p* = 0.004, η^2^ = 0.89] and d’ [*F*(2,24) = 12.57, *p* = 2E–4, η^2^ = 0.99]. *Post hoc* analysis showed that trained participants became faster and more accurate between the first and second repetition [*t*(12) = 3.15, *p* = 0.02, *d* = 1.82; *t*(12) = 4.21, *p* = 0.002, *d* = 2.43; for RT and d’, respectively] but no difference was found between the second and third repetition [*t*(12) = 1.24, *p* = ns; *t*(12) = 0.22, *p* = ns; for RT and d’, respectively]. This suggests that trained participants performance maxed out after the second repetition. On average, untrained participants increased their d’ recognition performance by 0.67 (from 0.52 ± 0.12 to 1.19 ± 0.21) and trained participants by 0.38 (from 2.47 ± 0.17 to 2.85 ± 0.22) between the first and the last repetition of the familiar images.

We found an effect of repetition for both groups of participants on d’. Learning of familiar images occurred over the course of the experiment for the untrained group (which could reflect medium-term memory, e.g., Melcher, 2001), but the same was not true for the trained group. Indeed, trained participants increased their performance only between the first and second session and for both d’ and RTs. This effect might be the consequence of learning a new task (recognition) compared to the task they used to perform in the training stage (categorization). Another hypothesis could be that a memory trace was reactivated after the first repetition of the stimulus. However, because both groups were presented with the stimulus set (study phase) before being tested in the recognition task, this memory trace could have already been reactivated (if needed) at this stage of the experiment.

Event-related-potential studies have shown that the early frontal difference between familiar and new items varies linearly with familiarity strength but is not sensitive to differences in amount of recollected experience associated with the items (Curran and Cleary, 2003; Woodruff et al., 2006; Yu and Rugg, 2010). In addition, the late parietal ERP component has been found to be correlated with the number of correctly retrieved contextual details (Vilberg et al., 2006; Vilberg and Rugg, 2009). Thus, the strength of familiarity might be reflected in the early frontal component while the amount of retrieved information migth be reflected in the late parietal component.

**FIGURE 7:**
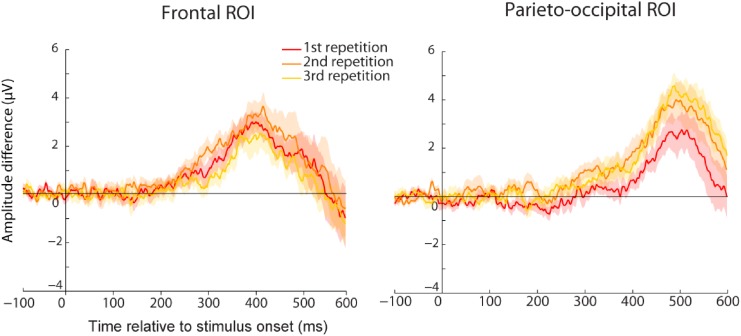
Average ERP amplitude difference between familiar and new images for the first (red), second (orange), or third (yellow) repetition of the familiar set. Results are represented for the trained group either for the frontal **(Left)** or the parieto-occipital **(Right)** ROI. Shared areas represent SEM. No significant difference was found between the repetitions although a trend was observed for the parietal ROI.

To assess any difference in the amount of familiarity or recollection, we compared the amplitude difference of the familiarity effect between the three repetitions for the frontal and parieto-occipital components that we identified earlier for the trained group (**Figure 7**). We found no evidence for larger familiarity strength at the end of the experiment (no significant difference between the three repetitions for the frontal ROI). However, even though not significant, we observed a trend for higher amplitude difference between the first and second repetition in the parieto-occipital ROI that miror behavioral findings. If confirmed, this parieto-occipital effect can be interpreted in two ways. First, since this component is believed to reflect the amount of retrieved information, it is possible that it represents additional image-related information being retrieved after the first repetition, which helped participants performing the task as reflected by their improved performance after the first repetition. On the other hand, it might also reflect a difference in motor decision or motor command, and this effect could represent increased participants’ performance after the first repetition.

### Effect of Stimulus Status in the Training Phase

The familiar image set was incidentally learned by the trained participants while they were performing an animal/non-animal task. There are many reasons to think that animal images could have been better encoded than non-animal images. First, since animal images were target during the training phase, participants might have paid more attention to them. Second, target images were linked to a specific concept (animal) while distractor images were not (non-animal images could be houses, city landscape, forests, etc.). For example, (Wiseman and Neisser, 1974) have showed that participants tested on ambiguous faces recognized stimuli judged as a face during the learning phase better than stimuli judged as a meaningless pattern. Finally, recent studies suggested that memory would have evolved in such way that animate stimuli would be better remembered than inanimate ones (Nairne et al., 2008; Nairne and Pandeirada, 2010a,b). This “animacy effect” has been found in recognition memory tasks involving words (Nairne et al., 2013; VanArsdall et al., 2013) and pictures (Bonin et al., 2013).

**FIGURE 8:**
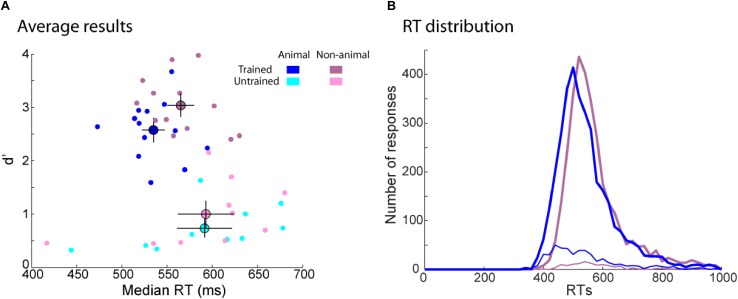
Behavioral results for animal and non-animal images separately in the recognition task. **(A)** d’ as a function of RT. Each dot represents one participant performance on animal (blue) and non-animal (purple) images. Average performance for each group (trained participants in dark color, untrained participants in light color) is represented by a bigger dot with SEM. **(B)** RT distribution for animal (blue) and non-animal (purple) images for the trained group. The number of correct (Hit: thick curves) and incorrect (FA: thin curves) responses were computed in a 20 ms bin.

To test this effect in our experiment we compared participants’ performance for animal and non-animal images (**Figure 8A**). RTs were similar between animal and non-animal images for the untrained group [*t*(9) = 0.14, *p* = ns] but trained participants responded around 34 ms faster to animal images [*t*(12) = 3.94, *p* = 0.002, *d* = 2.27]. Interestingly, RT distribution for non-animal images was shifted by around 30 ms for the earliest responses (400 ms) compared to animal images, but at longer latencies the two distributions overlapped (**Figure 8B**). Recognition performance (d’) was overall better for non-animal than animal images for the trained group [*t*(12) = 4.94, *p* = 3E–4, *d* = 2.85]. One might think that low performance for animal images might be an effect of accuracy trade-off. However, d’ was also better for non-animal images for the untrained participants [*t*(9) = 3.20, *p* = 0.01, *d* = 2.13] even though RTs were the same for both types of images. For the untrained and the trained group, animal images generated more responses in terms of false alarms (FA) [*t*(12) = 5.40, *p* = 2E–4, *d* = 3.12; *t*(9) = 4.66, *p* = 0.001, *d* = 3.11; for the trained and the untrained group, respectively] and Hits [*t*(12) = 3.08, *p* = 0.01, *d* = 1.78; *t*(9) = 2.20, *p* = 0.06, *d* = 1.47; for the trained and the untrained group, respectively]. Thus, there was overall a bias toward familiar responses for animal images. We develop below possible explanations of these results for (a) lower d’ on animal images and (b) faster RTs on animal images for trained participants compared to non-animal images.

### Lower d’ on Animal Images

Neither trained or untrained participants remembered animal images better than non-animal ones. Indeed we found the opposite effect. Compared to studies, which have found an animacy effect, we tested our participants in a go/no-go task and not in a free recall task. However, we think that the discrepancy with previous results might be better explained by the similarity between familiar and new images. It has previously been shown that the similarity within the familiar image set influences recognition memory performance (e.g., Koutstaal and Schacter, 1997; Konkle et al., 2010a; Huebner and Gegenfurtner, 2012). Recognition performance is worse if many stimuli of the same basic category have to be remembered than for very dissimilar stimuli. In our study, the overall number of responses (Hits and FAs) for animal images was higher than for non-animal images so participants were more likely to think that a new image of an animal (e.g., lion) was familiar since many lions were presented during the training phase. Indeed, images within the animal category were more similar than the ones within the non-animal category. In the first case, different kinds of the same basic animal category could be presented whereas in the second case, non-animal images were more diverse (they included plants, fruits, vegetables, fireworks, buildings, vehicles, desert, forest, etc…). In Bonin et al. (2013), the authors ruled out the possibility that differences between animate and inanimate objects was attributable to differences in the richness of perceptual or semantic features. However, they did not control for the amount of similarity between stimuli. Thus, it is possible that in their study animate stimuli were more distinctive than inanimate ones whereas the opposite was true in our experiment.

### Faster RTs on Animal Images for the Trained Group

**FIGURE 9:**
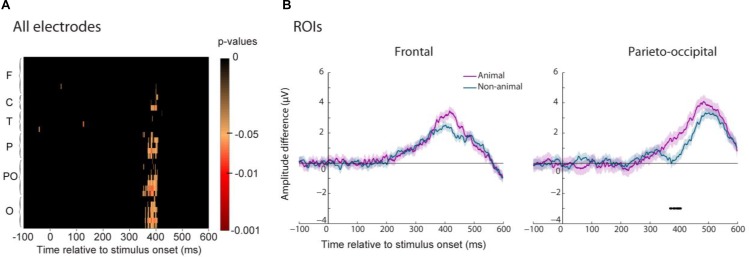
Differences between the ERP familiarity effect for animal and non-animal images for the trained group. **(A)** *P*-values (FDR-corrected) of this difference are represented for each electrode grouped by regions (same legend as in **Figure 5**). **(B)** The average amplitude difference of the familiarity effect is represented for animal (purple) and non-animal (blue) images for the two ROIs (frontal on the **Left** and parieto-occipital on the **Right**). Significant differences (*p* < 0.05) between animal and non-animal familiarity effect is reported by a line underneath the ERP.

Trained participants responded faster to animal than to non-animal images. We computed the ERP familiarity effect for the trained group separating animal and non-animal images and then compared these two familiarity effects. We performed this analysis on all electrodes and each time point as well as for the two ROIs (**Figure 9A**). We found a significant difference between the old/new effect for animal and non-animal images mostly visible in parietal, parieto-occipital and occipital regions. The stimulus status (animal or non-animal) had no significant effect on the early old/new effect in frontal regions (**Figure 9B**) even though higher amplitude was observed for animal images. This trend could be interpreted as higher familiarity strength for animal than for non-animal images. In parieto-occipital regions, the familiarity effect started around 30 ms later for non-animal images compared to animal images (370 ms vs. 405ms). If we consider the parieto-occipital effect as driven by recollection processes, then this shows that recollection of animal images was faster than the one for non-animal images. However, we cannot be sure about the nature of this component. Since we used a go/no-go protocol, this effect could also be driven by motor processes. Indeed, this 30 ms shift matches the one observed between the earliest RTs latencies for animal and non-animal images.

Thus, ERP analysis showed that the early stage of visual memory recognition (frontal component) was similar between animal and non-animal images. The familiarity effect started with the same latencies for both types of images (so on by contrast with the parieto-occipital component it cannot be linked to motor processes) and a non-significant trend for higher amplitude of the familiarity effect was observed for animal images, possibly because they were targets in the training phase (therefore participants remembered these images better). Later processes in the parieto-occipital region were faster for animal than for non-animal images, explaining faster RTs for animal images. These processes could be either explained by a faster access to details about the stimulus in long-term memory (recollection) or faster motor processes. This second explanation can be the consequence of the training procedure in which participants had to release their finger for animal images and inhibit their response for non-animal images. By repeating this categorization task, a higher fluency for animal images and/or an inhibition for non-animal images might have been set up.

### Animal vs. Non-animal Images

An ERP difference between animal and non-animal images starting around 150 ms after stimulus onset has been reported in many animal/non-animal categorization tasks (e.g., Thorpe et al., 1996; VanRullen and Thorpe, 2001). Moreover, Carlson et al. (2013) have showed that the category of an image can be decoded using EEG signal even though participants were not performing a categorization task. Here we tested whether we could find an ERP difference between animal and non-animal images while participants performed a familiar/non-familiar task.

**FIGURE 10:**
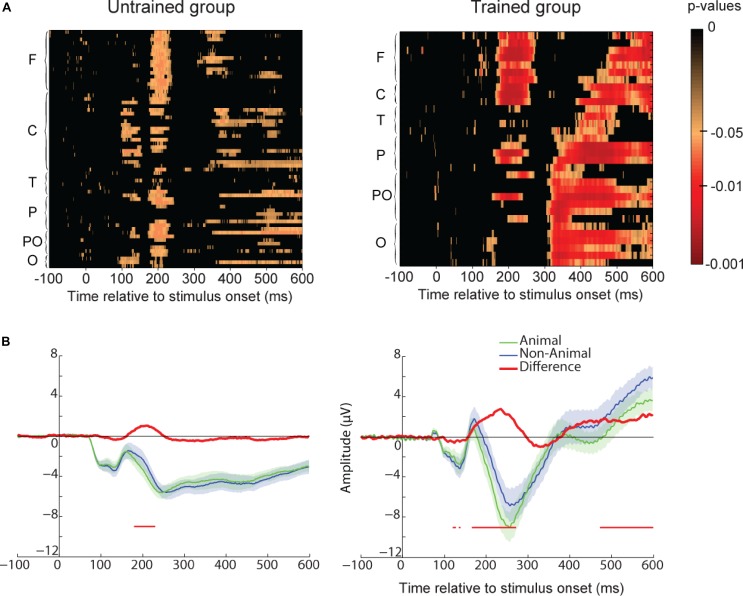
Animal/non-animal ERP différences in the recognition task for the untrained and trained group. **(A)** Paired *t*-test *p*-values (FDR-corrected) for the ERP difference between all animal and non-animal images. Same legend as in **Figure 5**. **(B)** ERP amplitude for animal and non-animal images for Frontal ROI. The difference is represented in red with significant *p*-values (*p* < 0.05) reported by a thin line below the ERP.

For both the trained and untrained participants, we compared the ERP elicited for an animal image to the one elicited for a non-animal image, regardless of whether the image was familiar or unfamiliar. The results showed a significant difference between these two conditions for both groups of participants (**Figure 10A**). This category effect was present mainly in frontal and central electrodes and was significant around 170 ms after stimulus onset even though it started around 150 ms (**Figure 10B**). Thus the stimulus category, animal or non-animal, was automatically processed while participants performed an unrelated task.

## Discussion

The main goal of our study was to test whether 20 ms flashed natural scenes could be stored in long-term memory. We tested two groups of participants: one group saw the 200 familiar images set only once and another group saw the same images several times over 3 weeks. Our results show that after only one viewing of 200 flashed images, participants can only remember 64% of them (in the first repetition) but if the same set of flashed images is repeated over 15 days in an unrelated task, participants can remember 87% of the images. These results are impressive for five raisons. First, trained participants were tested 1 week after the training phase so any recognized image had to be stored in long-term memory. Second, images were presented for only 20 ms, which avoid any possible eye movements and visual exploration during the encoding or the recognition of the images. Third, participants were not explicitly asked to memorize these images and did not know that they would be tested on a recognition task. Fourth, to reach that level of performance, participants had to remember enough detail of their familiar image set to be able to distinguish between two exemplars of the same basic category (e.g., between a familiar and a new lion). Fifth, participants were tested in a speeded categorization task (go/no-go paradigm) so they had to retrieve the image information quickly enough to be able to perform the task.

### Implicit Encoding in Long-Term Memory

Trained participants were able to recognize 87% of the images they had seen a week before in an animal/non-animal categorization task. It could be argued that this high level of performance was the consequence of the viewing the 200 images a few minutes before the experiment. In fact, when testing is delayed, recognition performance is usually lower than if the testing happens the same day as the training phase (e.g., Huebner and Gegenfurtner, 2012). However, untrained participants also saw the 200 images a few minutes before the recognition task but their performance were close to chance level (64%). Moreover, both groups of participants were not aware that they would be tested in a recognition task. Thus, even if the presentation of the images before the task induced a reactivation of a memory trace for the trained group, it still means that a memory trace was created during the categorization task performed a week before the recognition task.

In other studies in which pictures were incidentally learnt, recognition performance was higher than what we report here for the untrained participants. For example, using a similar living/non-living categorization task as a study phase, studies reported 70% (Groh-Bordin et al., 2005) or even 87% (Küper et al., 2012) correctly recognized objects. Even when the new images included new objects from the same category as familiar ones, participants’ recognition performance was still very high – e.g., 75% in Ecker and Zimmer (2009). However, there are two major differences between our studies and these studies. First, even when similar objects were used, only one stimulus per object category was included. Second, images were presented for a much longer time than in our study (from 500 to 2000 ms). Compared to the trained group, these two factors might have allowed better implicit encoding of the stimuli.

### Briefly Presented Images Can Be Memorized

Earlier studies on recognition memory have commonly used very long stimulus presentation times for the encoding and the testing phase. The fastest stimulus duration used was probably in the study of Huebner and Gegenfurtner (2012) in which stimuli were presented for 750 ms in the encoding phase with a 1s blank interval between images, and for 2000 ms in the testing phase with no time limit to respond. Here we used images flashed for only 20 ms which avoids any possible saccade that could help participants to encode additional details of the stimulus (Loftus, 1972; but see also Melcher and Kowler, 2001). Indeed even when we cumulate the presentation time of the images over the 21 repetitions before testing (420 ms), images were still presented for a shorter duration than in Huebner and Gegenfurtner (2012). However, the presentation of the images in our task was not followed by a mask. It is thus possible that participants processed the image until the next trial (around 1 s later). Indeed, Intraub (1979) showed that the time between two to-be-remembered pictures played a larger role in recognition memory performance than stimulus duration. In her study participants were presented with a stream of 16 pictures (RSVP paradigm) and were then tested on a recognition task. Stimulus onset asynchrony (SOA) varied from 110 ms (no blank interval between two pictures) to 1500 ms while presentation time of the pictures remained 110 ms. Participants were able to recognized 92.5% of the pictures with a 1500 ms SOA but only 25% of the pictures with a 110 ms SOA. In a following experiment, Intraub (1980) showed that recognition performance was similar for pictures showed for 110 ms followed by a 5890 ms blank interval (80% correct recognition) and for pictures showed for 6000 ms (94% correct recognition). Thus, brief stimulus presentation does not have a major importance on memory performance.

However, in these experiments participants knew that they would be tested in a recognition task before the presentation of the images so they could encode all necessary details to recognize them later on. In our study, participants had no reason to do so in the animal/non-animal categorization task used in the training phase. Furthermore in the studies of Intraub (1980) participants were tested only a few minutes later (compared to 1 week in our study) and could rely on medium-term memory (Melcher, 2001). Moreover, new images were very different than the familiar images as she used *“Distractors that did not bear a close resemblance to any of the stimuli were chosen”* (Intraub, 1980). Thus, the results of these experiments cannot be directly compared to ours. However, our results might fit better in the frame of the total time hypothesis (Bugelski, 1962; Cooper and Pantle, 1967). This theory suggests that the information stored in memory would depend on the total amount of time that a stimulus has been seen in one or several viewings. Our results are important in this framework since they show that this hypothesis could be applicable for stimuli presented over many days.

### How Detailed Are the Stored Representations?

The similarity of the familiar and the new image sets can influence memory. In recognition memory tasks, many studies have shown that participants were faster and more accurate to reject stimuli that were different to the familiar objects than to reject stimuli that were similar to them (Yago and Ishai, 2006; Wiesmann and Ishai, 2008). For example, increasing the number of scenes from similar semantic domain reduces memory for scene details (Melcher and Murphy, 2011). The effect of conceptual and perceptual similarity on recognition memory has been tested more systematically using pictures of objects (Konkle et al., 2010a) or scenes (Konkle et al., 2010b). In these studies, the authors varied the number of exemplars from each stimulus category that has to be remembered. They observed that performance decreased as the number of stored exemplars for each category increased and this effect was not predicted by perceptual distinctiveness of the exemplars but by conceptual distinctiveness (category) (see also Huebner and Gegenfurtner, 2012 who showed that both factors matter). In all these studies, recognition performance was very high. For example, even when 64 scenes from the same category had to be remembered, memory performance was at 76% accuracy (Konkle et al., 2010b). Altogether these results show that visual long-term memory depends on conceptual and possibly also visual similarity between the different stimuli to be stored.

In our study, we found that participants recognized animal images with lower accuracy performance than non-animal images. As mentioned in the results section, it is likely that animals were conceptually more similar than non-animal images thus leading to a higher number of false recognition. Indeed, half of the images were animals and some of the animals were from the same basic category (e.g., different images of lions). Moreover, compared to very high recognition performance observed in the studies mentioned above, untrained participants had low performance after only a single viewing of the images. These two observations suggest that the image set that we used required participants to be able to recognize enough details of the images to perform the task well. Remembering the gist of the image or the category of the object might not have been sufficient to perform the recognition task with the high accuracy performance observed for the trained participants.

### Automatic Object Categorization

Event-related-potential analyses revealed a difference between animal and non-animal images even though participants were performing a recognition task. We interpreted these results as an automatic access to object categories. This automatic access can be related to studies showing no requirements of focused attention to process the category of natural scenes (Li et al., 2002; Rousselet et al., 2004; Poncet et al., 2012). Dual tasks require attention (Tsuchiya and Koch, 2016), but focused attention cannot be used as both the central and peripheral stimuli are masked so that attention cannot be switched from one task to another. Another study using classification methods showed indirectly that information about stimulus category was present in EEG signal as soon as 120–130 ms after the onset of the stimulus presentation even when participants’ attention was focused on an unrelated task in the center of the screen while the object was presented in the background (Carlson et al., 2013). In our study, participants were asked to process the image but in order to determine its familiarity not its category. Nevertheless, we still found a significant effect of object category in ERP analyses from 170 ms after stimulus onset which is around the same time as reported in other studies (Fabre-Thorpe et al., 2001; VanRullen and Thorpe, 2001). As for the study of Carlson et al. (2013), we cannot be sure that object categories were not consciously processed. However, categorizing the object was irrelevant for the task and if participants were doing so, their reaction times should be longer than in a recognition task in which all stimuli are from the same category. Participants performed the recognition task at around 550 ms which is as fast as in studies using the same go/no-go paradigm using only faces (Ramon et al., 2011; Barragan-Jason et al., 2012). Altogether, these results argue in favor of an automatic access to object categories.

### Neuronal Correlates of Recognition Memory

Event-related-potential analyses showed a significant difference between familiar and new stimuli for the trained group. This familiarity effect started around 230 ms in frontal regions after stimulus onset and 300–400 ms in parieto-occipital regions which agrees previous studies reporting shorter latencies for pictures than for word recognition (Schloerscheidt and Rugg, 1997, 2004; Curran and Cleary, 2003). Curran and Cleary (2003) have observed an early (300–500 ms) mid-frontal component related to familiarity. We believe one reason for the earlier 230 ms difference we observed is due to the large number of stimuli (600 per category compared to 100 per category in Curran and Cleary, 2003) and the associated increase in statistical power. Many studies suggest that this differential activation in frontal and parietal cortex mediate two distinct memory processes, one for familiarity and one for recollection (Yonelinas, 1994; Curran, 2000; Yonelinas et al., 2005; Vilberg and Rugg, 2009; Curran and Doyle, 2011). However, other studies suggest that recollection and familiarity reflect differences in the strength of a common memory trace (Donaldson et al., 1996; Dunn, 2004; Gonsalves et al., 2005; Squire et al., 2007; Wixted, 2007; Wiesmann and Ishai, 2008). Nevertheless, in both hypotheses the prefrontal cortex and the posterior parietal cortex are involved in recognition processes. Indeed, these two regions respond more strongly in fMRI to stimuli judged as familiar compared to new (e.g., Rissman et al., 2010).

The meaning of the familiarity effect found in ERP studies is still under debate (e.g., Mecklinger et al., 2012; Paller et al., 2012). Different authors suggest that this effect might be the consequence of multiple components (e.g., novelty, familiarity, priming) that are difficult to detangle. For example, Tsivilis et al. (2001) suggested that the frontal effect was not a response to stimulus familiarity but to stimulus novelty. On the other hand, other studies argue that the ERP difference could be the consequence of the repetition of the familiar stimuli (conceptual priming) distinct from the neural correlates of familiarity (Voss and Paller, 2006; Paller et al., 2007; Voss et al., 2010; Voss and Federmeier, 2011). Against this hypothesis many studies argue that this effect varies with the perceptual overlap between stimuli at study and test (while conceptual processing remains unchanged) and that semantic priming (implicit retrieval) is located in more centro-parietal regions than the familiarity effect (explicit retrieval) (Ecker et al., 2007; Bridger et al., 2012; Küper et al., 2012; Lucas et al., 2012).

In our study, ERP were analyzed by including all trials. Thus, if there was any priming caused by the repetition of the images we should have been able to see it in both groups of participants. However, we found a familiarity effect only for the trained participants. This suggests that the ERP difference between familiar and new images reflected a process present for the trained group and absent in the untrained group. Furthermore, compared to priming studies, reaction times were not faster for familiar compared new items in an animal/non-animal categorization task (Fabre-Thorpe et al., 2001). Thus the early frontal component likely represents familiarity and not early categorical processing. Concerning the parieto-occipital component, our results show that it was tightly linked to the speed and accuracy of responses. Because our protocol involved a go response on familiar images and a no-go response on new images, we cannot rule out the possibility that it reflects motor activity rather than recollection processes. Indeed, one could argue that our participants did not recollect the images since they were only asked to perform a familiar/new task. However, the image set could include very similar objects and such difficult distinctions might therefore not be possible to perform solely based on familiarity. It is also possible that both motor and recollection components underlie the parieto-occipital effect that we observed.

### Effect Size and Statistical Power

The number of participants in this study is low – in particular for the untrained group (N-10). We report here power analyses performed post-oc on the data. Power analyses were performed using R “pwr” package. Our main behavioral results, showing that recognition performance was higher for trained than for untrained participants [*t*(21) = 8.52, *p* = 3E–8] yield an effect size of 3.52 and statistical power of 1. Our lowest effect size at 0.89 for image repetition effect yield statistical power of 0.8. For ERP analysis, considered independently of the fact that we used correction for multiple comparison, yield statistical power of 0.94 for the trained group (**Figure 5**), which means that the likelihood of probability of finding an effect that is present was 94%. Despite a reduced number of participants, large effects observed in our study indicate that statistical power is appropriate.

## Conclusion

Our results shows that detailed information about complex natural scenes flashed for only 20 ms can be implicitly stored in long-term memory. Participants were able to recognize familiar images if they had seen them several times in an unrelated task but not if they had seen them only once. This might be possible because the stimuli were repeated over many days in a categorization task and not just passively viewed. Our study highlights two functions that the brain seems to do without any top-down influence. First, information about object categories seems to be processed even though there is no requirement for it. Second, detailed encoding of visual stimuli happens without any explicit instruction.

## Author Contributions

MP and MF-T analyzed the data. AD, MF-T, and MP wrote the paper.

## Conflict of Interest Statement

The authors declare that the research was conducted in the absence of any commercial or financial relationships that could be construed as a potential conflict of interest.

## Supplementary Material

FIGURE S1 Paired *t*-test *p*-values false discovery rate (FDR-corrected) for the event-related-potential (ERP) difference between go and no-go responses for the untrained group considering only the first repetition (although the untrained group did see these image during the study accuracy was at chance level) **(A)** or the entire testing phase **(B)**. The difference is represented at each time point from −100 to 600 ms relative to stimulus onset. Electrodes are grouped by regions as in **Figure 5**.
