# Supplementary figures and images for: Briefly Flashed Scenes Can Be Stored in Long-Term Memory
Source: Front Neurosci. 2018 Oct 5;12:688. doi: 10.3389/fnins.2018.00688 (PMC6182062; doi:10.3389/fnins.2018.00688)

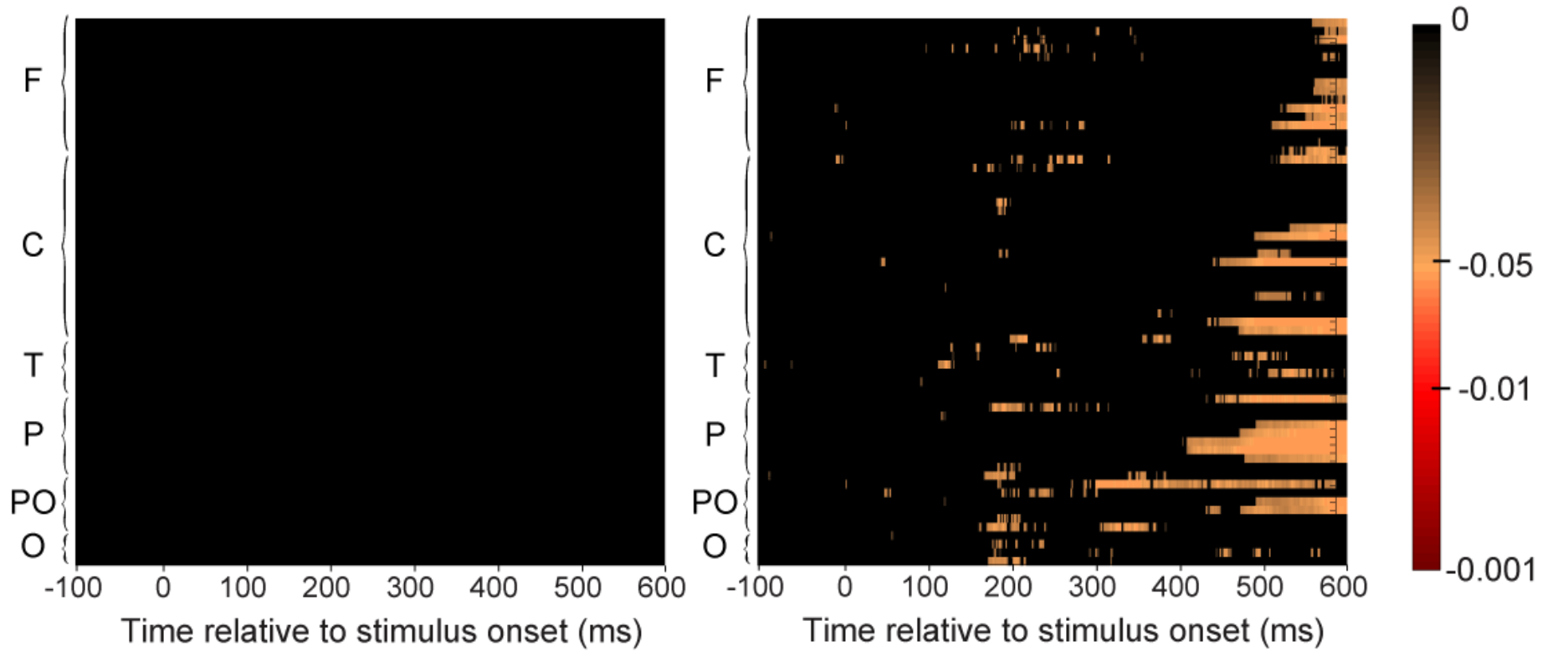

Supplement: FIGURE S1 — Paired t-test p-values false discovery rate (FDR-corrected) for the event-related-potential (ERP) difference between go and no-go responses for the untrained group considering only the first repetition (although the untrained group did see these image during the study accuracy was at chance level) (A) or the entire testing phase (B). The difference is represented at each time point from −100 to 600 ms relative to stimulus onset. Electrodes are grouped by regions as in Figure 5. [file Image_1.png]
